# Cerebral nocardiosis in a patient treated with pembrolizumab: a first case report

**DOI:** 10.1186/s12879-022-07288-4

**Published:** 2022-03-29

**Authors:** Paul Petitgas, Mathieu Lesouhaitier, Sarrah Boukthir, Vincent Cattoir, Pierre Tattevin, François Bénézit

**Affiliations:** 1 Infectious Diseases and Intensive Care Unit, Pontchaillou University Hospital, Rennes, France; 2 Infectious Diseases and Internal Medicine Unit, Saint-Pierre University Hospital, La Reunion, France; 3 Clinical Microbiology Laboratory Unit, Pontchaillou University Hospital, Rennes, France

**Keywords:** Nocardiosis, Pembrolizumab, IRAE, Case report

## Abstract

**Background:**

Checkpoints inhibitors (CPIs) are increasingly used for the treatment of several malignancies. The most common side effects are Immune Related Adverse Events, while infectious complications are rare, especially cerebral nocardiosis.

**Case presentation:**

Here, we report the first clinical case of a cerebral nocardiosis revealed after seizure in a patient treated by pembrolizumab for a metastatic lung cancer, in the absence of any additional immunosuppressive therapy or risk factors for cerebral nocardiosis. The extended evaluation including a brain CT-scan did not reveal any lesion before pembrolizumab. Nevertheless, the 3-month delay between the start of Pembrolizumab and the diagnosis of cerebral nocardiosis suggests that the infection occurred prior to the CPI. Unfortunately, the patient died during treatment for cerebral nocardiosis, while the lung cancer tumor mass had decreased by 80% after the sixth cycle of pembrolizumab.

**Conclusions:**

This case report emphasizes that clinicians should consider diagnoses other than metastasis in a patient with a brain mass and metastatic cancer treated with CPI, such as opportunistic infections or IRAE.

## Background

By enhancing natural immunity against malignancies, immune checkpoints inhibitors (CPIs) have opened a new era in the treatment of cancer [1–4]. These CPIs include anti-CTLA-4 (cytotoxic T-lymphocytes antigen 4), anti-PD-1 (programmed cell death 1) and anti-PD-L1 (programmed cell death-ligand 1) antibodies. They are approved for the treatment of various solid cancers (metastatic melanoma, non-small cell lung cancer, renal cell carcinoma, head and neck squamous cell cancer and urothelial carcinoma), and Hodgkin lymphoma.

These treatments upregulate the immune system, which may be associated with immune-related adverse events (IRAEs). These toxicities can affect multiple organs (skin, liver, kidney, digestive tract), and are often managed with corticosteroid therapy [5]. Infectious complications have been reported in less than 10% of patients receiving CPI, primarily common bacterial infections [6]. We report the first case of cerebral nocardiosis in a patient treated by a CPI.

## Case presentation

**Fig. 1:**
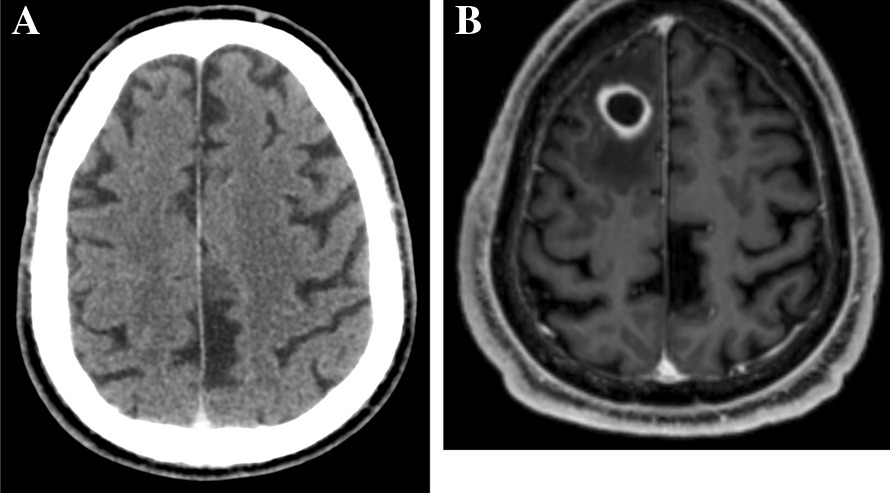
Brain CT before pembrolizumab start, and brain MRI at the time of cerebral nocardiosis diagnosis. **A** Brain CT before pembrolizumab start (no abnormality). **B** T1-weighted gadolinium-enhanced brain MRI at the time of cerebral nocardiosis diagnosis: 18 mm right frontal lobe round-shaped lesion with perilesional edema and wall enhancement

A 65-year-old-man was admitted in the emergency department for seizures. His medical history included diabetes mellitus, arterial hypertension, and smoking. Four months before admission, a chest X-Ray performed because of a persistent cough revealed a lung mass. The lung biopsy yielded a squamous non-small-cell lung cancer with 80% expression of PD-L1. Extended evaluation including a brain CT (Fig. 1A), thoracic CT, abdominal MRI and whole-body PET-scan showed a unique hepatic metastasis. Following consultation with the institutional multidisciplinary team in charge of lung cancer, the patient was treated with intravenous pembrolizumab (anti-PD-1 antibody), started 3 months before admission, as a single-drug regimen, 200 mg every three weeks, with no tolerability or adherence issues.

On admission, the patient was afebrile, and clinical examination was normal. Routine biochemical and hematology tests were normal, with blood leukocytes count of 7.8 G/l, and serum C reactive protein at 1.1 mg/l. The electroencephalogram performed 12 h after seizures was normal. Brain CT scan found a focal hypodense lesion in the right frontal lobe, further characterized by brain MRI as an intracranial round-shaped lesion with a maximal diameter of 18 mm, low signal intensity on T1-weighted and intermediate signal intensity on T2-weighted images, associated with perilesional edema and wall enhancement on T1-weighted gadolinium-enhanced images (Fig. 1B), suggestive of brain abscess, while brain metastasis could not be ruled out. The patient was transferred to the neurosurgical department for a total removal of the lesion (excision with craniotomy), both for diagnostic and therapeutic purposes. Per-operative macroscopic findings were also suggestive of brain abscess, and empirical antimicrobial therapy was started after surgery with a combination of intravenous ceftriaxone and metronidazole. Microscopic examination revealed numerous filamentous-branching Gram-positive rods. Cultures yielded *Nocardia farcinica*, identified by MALDI-TOF mass spectrometry. Antibiotic treatment was modified to meropenem 1 g every 8 h associated with cotrimoxazole 1600 mg/400 mg every 8 h and folinic acid. Body-CT did not show any other localization of nocardiosis, while the mass tumor had decreased by 80% after the sixth cycle of pembrolizumab. Drug susceptibility testing found a meropenem MIC of 32 mg/L, while linezolid MIC was 4 mg/L, and cotrimoxazole MIC was 0.5 mg/L. Meropenem was replaced by linezolid 600 mg every 12 h, in combination with cotrimoxazole. Following the diagnosis, additional risk factors for cerebral nocardiosis were ruled out.

After 2 months, the patient was asymptomatic, except for asthenia. Antibiotics were well tolerated. He did not receive additional dose of pembrolizumab. At 10 weeks of treatment, he developed thrombocytopenia due to linezolid. He died 3 months after the diagnosis of cerebral nocardiosis, because of a major upper-gastrointestinal bleeding probably related to a peptic ulcer disease and thrombocytopenia.

## Discussion and conclusions

To our knowledge, we report the first case of a cerebral nocardiosis in a patient treated by CPI. *Nocardia spp* are a rare cause of cerebral abscess, primarily among immunocompromised patients. Main risk factors include immunosuppressive drugs, cancer, organ transplant, HIV infection, anti-GM-CSF antibodies, and uncontrolled diabetes mellitus [7]. Cerebral nocardiosis has also been rarely reported in patients with no identified risk factors [8]. In the observation reported herein, the patient had been recently diagnosed with a metastatic lung cancer, a known risk factor for cerebral nocardiosis, but he did not receive any immunosuppressive treatment, and had no clinical or imaging features suggestive of cerebral nocardiosis before pembrolizumab was initiated. As a limitation, anti-GM-CSF were not investigated.

CPIs are not considered as risk factors for opportunistic infection. A retrospective study of 740 patients treated by CPI for a metastatic melanoma found that 7.3% presented at least one episode of serious infection [6]. Among them, 79% had a bacterial infection. Patients treated with pembrolizumab were at lower risk of infections as compared to patients treated with other CPIs. Of note, the concomitant use of immunosuppressive agents, especially infliximab and corticosteroids was the main risk factor for serious infections in that cohort, while these drugs are often used for the control of IRAE associated with CPI. Less common infections have been reported in patients treated with CPI, including tuberculosis reactivation [9], invasive aspergillosis, pneumocystosis or cytomegalovirus disease [10]. However, these complications have mostly been reported in patients with concomitant use of immunosuppressive agents, primarily corticosteroids for the management of IRAE. Hence, the role of CPIs in these opportunistic infections remains hypothetical. In our observation, the development of cerebral nocardiosis after 3 months of pembrolizumab treatment could also be related to IRAE, with a pathophysiology similar to the unmasking immune reconstitution inflammatory syndrome reported in HIV-infected patents: this hypothesis would imply that cerebral nocardiosis was already evolving by the time pembrolizumab was initiated, but was not identified despite the extensive diagnostic workout performed for cancer staging, in the context of an immune system altered by metastatic cancer. Following the dramatic oncologic response to pembrolizumab, the patient immune system recovered, and cerebral nocardiosis would hence be the manifestation of immune reconstitution. Equally plausible is that pembrolizumab enhanced anti-inflammatory immune responses and this led to manifestation of cerebral nocardiosis, or even pembrolizumab had no impact on the development of nocardiosis.

Whatever the mechanisms behind this severe adverse event, this case report outlines that the investigations for a cerebral mass in a patient with metastatic cancer treated with CPI should not be restricted to cerebral metastases, and also include opportunistic infections, as well as IRAE.

## Data Availability

The data used during the study are available from the corresponding author.

## Abbreviations

CPI: Checkpoint inhibitor
CTLA-4: Cytotoxic T lymphocytes antigen 4
PD-1: Programmed cell death 1
PD-L1: Programmed cell death ligand 1
IRAE: Immune-related adverse event
