# Correction to: Cerebral nocardiosis in a patient treated with pembrolizumab: a first case report

**DOI:** 10.1186/s12879-022-07343-0

**Published:** 2022-04-12

**Authors:** Paul Petitgas, Mathieu Lesouhaitier, Sarrah Boukthir, Vincent Cattoir, Pierre Tattevin, François Bénézit

**Affiliations:** 1 Infectious Diseases and Intensive Care Unit, Pontchaillou University Hospital, Rennes, France; 2 Infectious Diseases and Internal Medicine Unit, Saint-Pierre University Hospital, La Reunion, France; 3 Clinical Microbiology Laboratory Unit, Pontchaillou University Hospital, Rennes, France

## Correction to: BMC Infectious Diseases (2022) 22:306 10.1186/s12879-022-07288-4

Following publication of the original article [1], the authors identified that the given names and family names were erroneously transposed.

The incorrect authors’ names list is:

Petitgas Paul^1,2*^, Lesouhaitier Mathieu^1^, Boukthir Sarrah^3^, Cattoir Vincent^3^, Tattevin Pierre^1^ and Bénézit François^1^.

The correct authors’ names list is:

Paul Petitgas^1,2,*^, Mathieu Lesouhaitier^1^, Sarrah Boukthir^3^, Vincent Cattoir^3^, Pierre Tattevin^1^ and François Bénézit^1^.

The author group has been updated above and the original article [1] has been corrected.
